# Pernicious Anemia Presenting as Non-ST-elevated Myocardial Infarction and Depression

**DOI:** 10.7759/cureus.4870

**Published:** 2019-06-10

**Authors:** Hassaan Iftikhar, Maryam Saleem, Anand Kaji

**Affiliations:** 1 Internal Medicine, St. Francis Medical Center, Seton Hall University, Trenton, USA; 2 Internal Medicine, Waterbury Hospital, Waterbury, USA

**Keywords:** nstemi, depression, depression, pernicious anemia, b12, atrophic gastritis

## Abstract

Pernicious anemia (PA) is a common cause of megaloblastic anemia throughout the world, especially in Northern European whites. This disease is characterized by the deficiency of vitamin B12 due to the presence of anti-intrinsic factor and anti-parietal cell antibodies which inhibit the absorption of the vitamin B12. In cases of severe vitamin B12 deficiency, patients can suffer debilitating complications such as described in our case.

## Introduction

Vitamin B12 is a coenzyme required for deoxyribonucleic acid (DNA) synthesis [1], and its deficiency can lead to erythropoietic hemolysis in bone marrow, the formation of macrocytes, and hypersegmentation of neutrophils. Most cases of vitamin B12 deficiency are due to malabsorption rather than dietary deficiency. It can present with symptoms of anemia and/or neurologic dysfunction. It is a critical diagnosis as it is a reversible cause of anemia and can prevent transfusions. We describe an unusual case of vitamin B12 deficiency anemia presenting as non-ST-elevated myocardial infarction (NSTEMI) with depressive symptoms

## Case presentation

This study includes a 73-year-old female with the past medical history of hypertension, hypothyroidism, and coronary artery disease (previous cardiac catheterization showed 99% stenosis in the right coronary artery and stent placement in the circumflex artery) who presented with chest pain for one day. She described the chest pain as constant, progressive, non-radiating, 5/10 in intensity, and pressure-like in character with no aggravating or alleviating factors. She endorsed associated symptoms of increasing shortness of breath and generalized weakness. Her walking capacity had decreased from two blocks at baseline to 15 steps. She also described her diet mainly being as tea, crackers, canned food, bread, and cheese. The review of systems was significant for depressed mood and low energy. She denied any history of gait disturbances, weakness, memory loss, dizziness, lightheadedness, syncope, cough, difficulty in maintaining posture, numbness or tingling in feet, bleeding, and any gastrointestinal surgery. Family history was significant for laryngeal carcinoma in the father. The social history was significant for smoking one pack per day for 20 years which she had quit 30 years ago, and she denied any alcohol or illicit drug use. She was not taking any home medications.

Her physical exam on admission including vitals was normal except for the remarkable findings of pale conjunctiva and shiny tongue.

Her complete blood count is described in Table 1.

**Table 1:** Complete blood count

Test	Result	Reference range
Complete Blood Count		
Hemoglobin (Hb)	4.9 g/dl	12-16 g/dl
Hematocrit (HCT)	13.9 %	37-47%
Mean corpuscular volume(MCV)	142.2 fL	78-102 fL
Red cell distribution width (RDW)	24.4%	11.5-14.5%
Platelets	66000 thou/L	130-400 thou/L
Corrected Reticulocyte count	1.4%	
White blood cells (WBC)	5.2 thou/L	4.8-10.8 thou/L

The iron panel is described in Table 2.

**Table 2:** Iron panel

Test	Result	Reference range
Iron Panel		
Serum Iron	37 mg/dl	37-145 mg/dl
Transferrin	138 mg/dl	200-360 mg/dl
Ferritin	144 mg/dl	13-150 mg/dl
Transferrin saturation	19 %	

Other miscellaneous laboratory findings are given in Table 3.

**Table 3:** Miscellaneous laboratory results

Test	Result	Reference range
Miscellaneous		
Vitamin B12 level	35 pg/ml	211-946 pg/ml
Folic acid	16 ng/ml	>4.7 ng/ml
Lactate dehydrogenase (LDH)	1444 U/L	135-214 U/L
Methylmalonic acid level	>5.00 umol/L	<0.4 umol/L
Total Bilirubin	1.5 mg/dl	<1 mg/dl

On further workup, her peripheral smear showed hypersegmented neutrophils, the intrinsic factor antibody was negative, but the parietal cell antibody was positive. Troponins were elevated at 0.78 followed by 0.92. EKG showed new onset T-wave inversions in lead I, aVL, V5, and V6. 2D echo revealed a drop in the EF from 55% to 25% in two years. As such, she was diagnosed with NSTEMI and depressed mood likely secondary to pernicious anemia (PA).

Over the hospital course, the patient received three units of red blood cells and daily intramuscular injections of 1000 micrograms of cyanocobalamin which improved the hemoglobin to 8.6 g/dl and decreased the MCV to 103.5. Her chest pain resolved significantly and her depressive symptoms were ameliorated. The plan on discharge was to give the patient vitamin B12 shots weekly and outpatient esophagogastroduodenoscopy (EGD) with monitoring of serial CBCs. The patient was made appointments to follow up with her gastroenterologist, hematologist, and primary care physician.

## Discussion

PA is the most common cause of megaloblastic anemia throughout the world especially in Northern European whites but the incidence is also increasing in non-northern European or Africans. Multiple cases of PA associated with autoimmune hemolytic anemia have been described in the literature [1-2]. However, there have not been cases of PA leading to NSTEMI in the literature. Vitamin B12 is found in animal meat, shellfish, liver, and dairy. The normal absorption of vitamin B12 involves binding to intrinsic factor (IF) and getting absorbed in the terminal ileum. However, in PA, patients have anti-parietal cell antibodies (Ab) and/or anti-IF Ab, which prevent the production of IF and inhibit B12 binding to IF and absorption, respectively [2-3]. The tea and toast diet of old age can exacerbate the deficiency. However, most cases of vitamin B12 are due to malabsorption rather than a dietary deficiency. At the level of bone marrow, vitamin B12 deficiency causes ineffective erythropoiesis leading to the formation of megaloblasts, hypersegmented neutrophils, and early destruction of erythroblasts by apoptosis causing elevated lactate dehydrogenase (LDH), bilirubin, and low reticulocyte count [1]. Pancytopenia may occur as well. PA is associated with autoimmune atrophic gastritis which has a potential of causing intestinal type gastric metaplasia. For diagnostic purposes, an important point to note is that the low serum vitamin B12 levels are not as helpful as serum methylmalonic acid levels which are closer to representing tissue vitamin B12 levels [4]. Anti-IF Ab is highly specific but not sensitive but anti-parietal cell antibodies are not as sensitive or specific [5-6].

In addition to the hematologic manifestations, PA can have neurologic symptoms including but not limited to subacute combined degeneration of spinal cord, demyelination of dorsal column leading to ataxia, peripheral neuropathy, and psychiatric complications [7]. Also, it has been reported in the literature that the degree of bone marrow suppression is inversely related to neurologic symptoms and significant anemia rarely presents in coexistence with neurologic symptoms [8-9].

The pathophysiology of myocardial ischemia in the setting of vitamin B12 deficiency would be caused by the mismatch in myocardial oxygen supply and demand from the developing anemia in face of low B12. This is especially true for patients with atherosclerotic coronary artery disease in which severe anemia can present as chest pain and decreased exercise tolerance [5]. This gives the picture of type II myocardial infarction which is typical of demand ischemia [10]. Our patient presented with a unique complication of PA which has not been reported in literature earlier. Patients can be treated with oral vitamin B12 in doses of 1000-2000 ug or parenteral forms by 50-100 ug once weekly until the deficiency is corrected and afterward, monthly injections [5,11].

## Conclusions

Our case describes a unique scenario where PA led to myocardial ischemia in the absence of any other cause. It also lays emphasis on the importance of early recognition of PA, especially in the elderly population who are at higher risk due to tea and toast diet in addition to autoimmune pathophysiology. It also describes the treatment of PA and the potential downstream complications such as myocardial infarction and depression which might develop if the condition goes undiagnosed for a long time.
